# Changes in Mitochondrial Epigenome in Type 2 Diabetes Mellitus

**DOI:** 10.3389/bjbs.2023.10884

**Published:** 2023-02-14

**Authors:** Hui Ching Low, William M. Chilian, Wickneswari Ratnam, Tilakavati Karupaiah, Mohd Fairulnizal Md Noh, Fazliana Mansor, Zhi Xiang Ng, Yuh Fen Pung

**Affiliations:** ^1^ Division of Biomedical Science, Faculty of Science and Engineering, University of Nottingham Malaysia, Semenyih, Selangor, Malaysia; ^2^ Integrative Medical Sciences, Northeast Ohio Medical University, Rootstown Township, OH, United States; ^3^ Department of Biological Sciences and Biotechnology, Faculty of Science and Technology, Universiti Kebangsaan Malaysia, Bangi, Selangor, Malaysia; ^4^ School of Biosciences, Faculty of Health and Medical Sciences, Taylor’s University Lakeside Campus, Subang Jaya, Selangor, Malaysia; ^5^ Nutrition, Metabolism and Cardiovascular Research Centre, Institute for Medical Research, National Institute of Health, Setia Alam, Shah Alam, Malaysia; ^6^ School of Biosciences, Faculty of Science and Engineering, University of Nottingham Malaysia, Semenyih, Selangor, Malaysia

**Keywords:** mitochondrial DNA, methylation, DNMT, D-loop, copy number

## Abstract

Type 2 Diabetes Mellitus is a major chronic metabolic disorder in public health. Due to mitochondria’s indispensable role in the body, its dysfunction has been implicated in the development and progression of multiple diseases, including Type 2 Diabetes mellitus. Thus, factors that can regulate mitochondrial function, like mtDNA methylation, are of significant interest in managing T2DM. In this paper, the overview of epigenetics and the mechanism of nuclear and mitochondrial DNA methylation were briefly discussed, followed by other mitochondrial epigenetics. Subsequently, the association between mtDNA methylation with T2DM and the challenges of mtDNA methylation studies were also reviewed. This review will aid in understanding the impact of mtDNA methylation on T2DM and future advancements in T2DM treatment.

## Introduction

Diabetes mellitus (DM) is a chronic metabolic disorder typified by the presence of hyperglycaemia (1, 2). Globally, prevalence of DM continues to increase by the millions every year, as of 2021, 537 million adults were diagnosed with DM (3). DM patients can be further classified into subsets based on the aetiology. Type 2 DM (T2DM) is the most diagnosed class of DM, accounting for 90%–95% of all cases (4).

T2DM is mainly characterised by insulin resistance, with multiple pieces of evidence showing the key role of mitochondria involvement (5–8). Due to the central role of mitochondria in multiple cellular responses and signalling pathways, mitochondrial dysfunction will influence the development and progression of T2DM (9). Thus, information on the association between mitochondria dysfunction and T2DM is of interest to public health. Factors that may regulate mitochondrial dysfunction such as mitochondrial epigenetics (influenced by external factors like lifestyle intervention) are important to the understanding and treatment of T2DM. Till date, several studies have established the connection between mitochondrial epigenetics and T2DM, but the exact effect of mitochondrial epigenetics on T2DM remains elusive (10–12).

The objectives of this review are to provide an overview on mitochondrial epigenetics, to address the association between mitochondrial epigenetics with T2DM and to conclude by discussing the challenges of mitochondrial epigenetics studies.

## Epigenetics

The expression of genes is known to depend on the genetic sequence and epigenetic regulation of the gene. Epigenetics refers to the study of inheritable changes which would alter gene expression either transiently or permanently without permanent modifications to the original DNA sequence (13, 14). Due to its unique and flexible nature, epigenetics presents a deeper insight into the varied gene expression profiles of individuals sharing the same genetic sequence (15).

**FIGURE 1:**
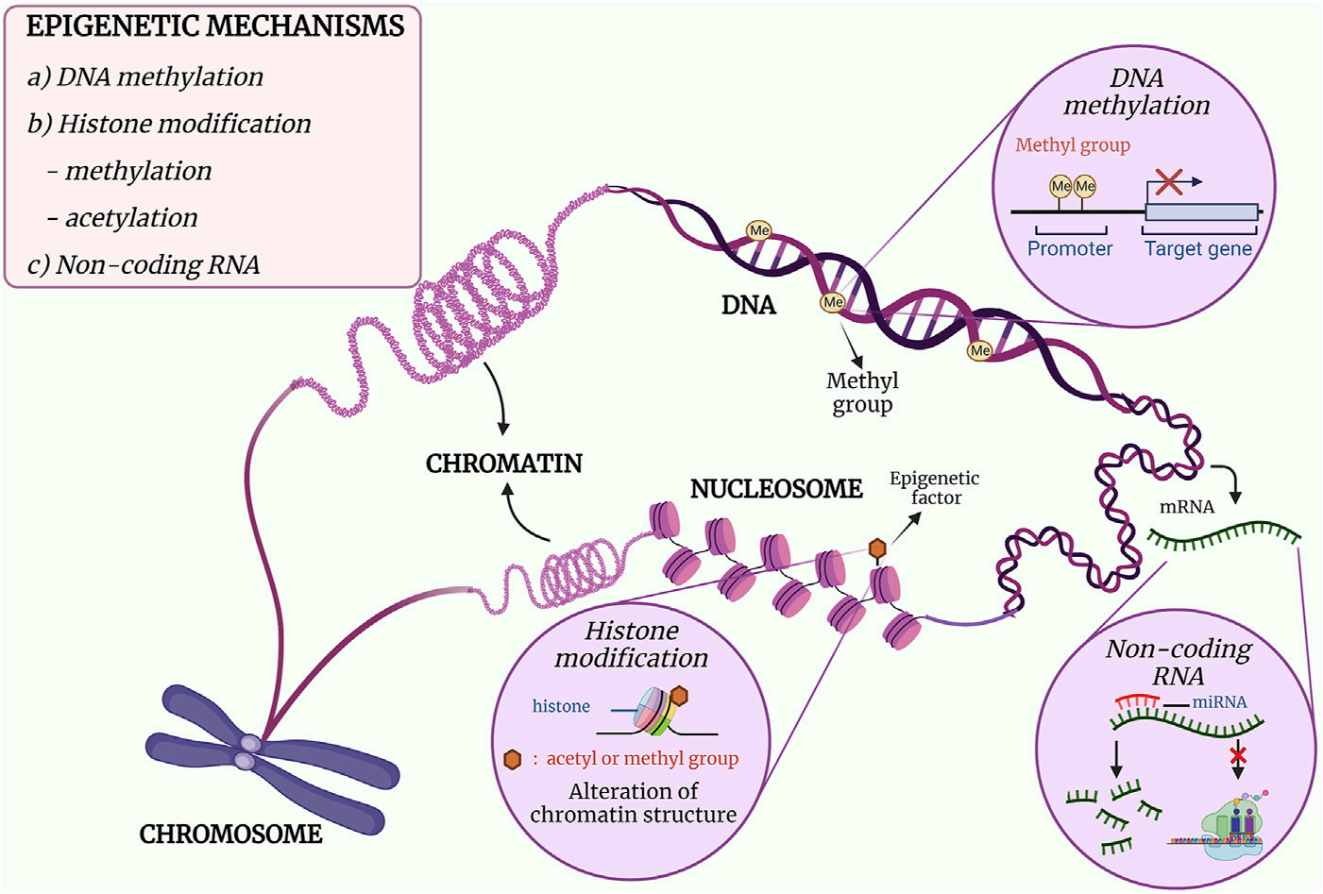
The three major mechanisms of epigenetics. (a) DNA methylation is the formation of a methylated cytosine through the transfer of a methyl group to the fifth carbon of the cytosine residue. When methylation occurs at the promoter region, downregulation of gene expression occurs. (b) The post-translational covalent addition of an acetyl or methyl group at the histone would alter the chromatin structure, resulting in regulation of gene expression. (c) The binding of a non-coding RNA such miRNA to mRNA would either inhibit the translational activity of the mRNA or degrade the mRNA. Adapted and modified from (19, 20).

The mechanisms of epigenetics can be mainly categorised into three classes, namely, DNA methylation, post-translational modifications (PTMs) of histones and gene expression regulation by non-coding RNAs (ncRNAs) (16–18). DNA methylation is the covalent addition of a methyl group to the cytosine leading to inhibition of gene expression, while PTMs of histones are the addition of an acetyl or a methyl group that would alter the chromatin structure. As for ncRNAs, it acts post-transcriptionally in which it binds to a messenger RNA (mRNA) and either degrade the mRNA or inhibit protein translation (16). The mechanism and effect of the epigenetic modifications are illustrated in Figure 1. In essence, each class plays its role in the regulation of gene expression, and DNA methylation will be discussed in detail in this review.

### Nuclear DNA Methylation

**FIGURE 2:**
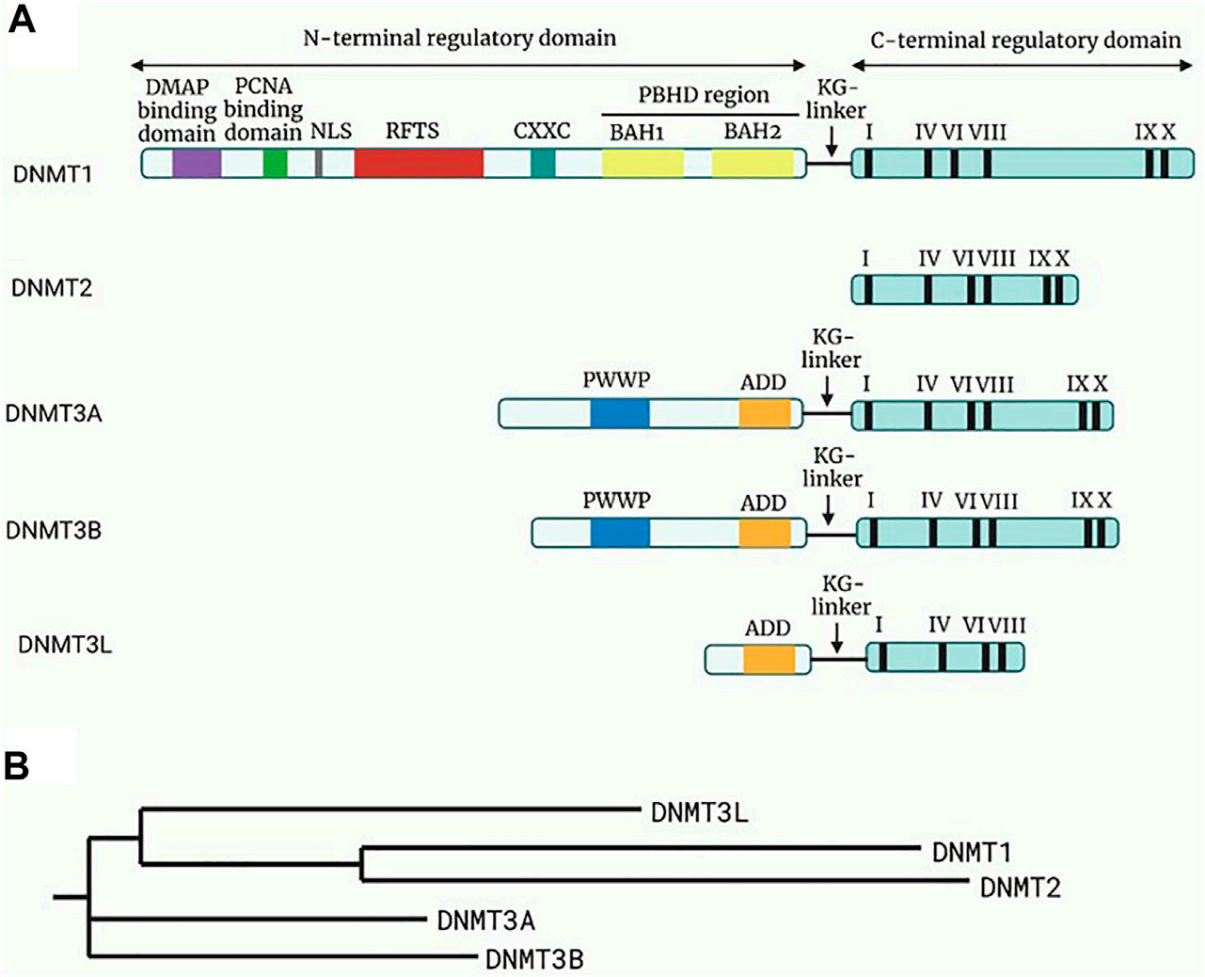
Structure and the phylogeny of known DNA methyltransferases (DNMTs). **(A)** A schematic diagram of the structure of known DNMTs with their respective domains. The N-terminal regulatory domain is linked to the C-terminal regulatory domain through a KG-linker consisting of lysine and glycine residues. DMAP will bind to DMAP binding domain in DNMT1 leading to repression of transcription. While the function of the remaining domain on the N-regulatory domain are not fully elucidated, it is known that the roman numerals in the C-terminal represents conserved motifs responsible for the catalytic activity of DNMTs. Adapted and modified from (24). **(B)** A phylogenetic tree of the DNMTs constructed through Neighbour-joining method. Reference sequences of DNMTs were obtained from UniProt database with accession number: DNMT1 (UniProt ID: P26358), DNMT2 (UniProt ID: O14717), DNMT3a (UniProt ID: Q9Y6K1), DNMT3b (UniProt ID: Q9UBC3), DNMT3l (UniProt ID: Q9UJW3). Abbreviations: DMAP, DNA methyltransferase associated protein; PCNA, Proliferating cell nuclear antigen; NLS, Nuclear localisation signal; RFTS, Replication foci targeting sequence; CXXC, Cysteine-rich zinc ion binding domain; PBHD, Polybromo homology domain made of two bromo-adjacent homology (BAH); PWWP, Pro-Trp-Trp-Pro conserved motif; ADD, Cysteine-rich domain.

Methylation of nuclear DNA (nDNA) typically involves the addition of a methyl group (CH_3_) from the S-adenosyl methionine to the fifth carbon (C5) of the cytosine residues, which are paired with guanine bases (CpG), leading to the formation of 5-methylcytosine (5mC) and the by-product S-adenosylhomocysteine (21). However, it was also identified at non-CpG sites such as CpA, CpT and CpC sites (22, 23). The methylation pattern is mainly established and regulated by a group of enzymes collectively termed DNA methyltransferases (DNMTs), as depicted in Figure 2. In this group, only DNMT1, DNMT3a and DNMT3b were found to be involved in the overall process of methylation.

DNMT1 was proposed to be the primary enzyme responsible for maintaining normal methylation patterns by restoring hemimethylated sites in CpG sequences to fully methylated sites during replication (25, 26). It was suggested that this may have risen from its preference for hemimethylated DNA, localisation at replication foci, and the ability to process long stretches of DNA (27). Meanwhile, *de novo* methylation at the unmethylated CpG sequences is mediated by DNMT3a and DNMT3b during embryogenesis, in which the PWWP domain ensures their binding to DNA (23, 26). Table 1 shows the summarised role of each DNMT in methyltransferase activity.

**TABLE 1:** Structure and function of DNA methyltransferases (DNMTs). Adapted from (26-29).

Component		DNMT1	DNMT2	DNMT3A	DNMT3B	DNMT3L
Domain structure	N-terminal	Present	Absent	Present	Present	Present
	C-terminal	Present with slight differences for each DNMT				
Enzymatic activity		Active				Inactive
Methyltransferase profile	Methylation sites	DNA	tRNA	DNA	DNA	DNA
	Roles	• Maintains methylation patterns during DNA replication and cell proliferation	• Initiates methylation of the tRNA of aspartic acid at the 38 cytosine of the anticodon loop	• Initiates methylation in a distributive manner	• Initiates methylation in a processive manner	• Acts as a regulatory factor and mediates methylation activity of DNMT3
		• Localises at replication foci and able to process long stretches of DNA		• DNMT3A1 is predominant in heterochromatic regions of differentiated cells	• Acts as accessory protein in methylation activity	• Express in germ cells and embryonic cells only
				• *De novo* activity is inhibited by BAH domain	• DNMT3A2 is predominant in euchromatic regions of undifferentiated cells		

BAH domain, Bromo-adjacent homology domain.

**FIGURE 3:**
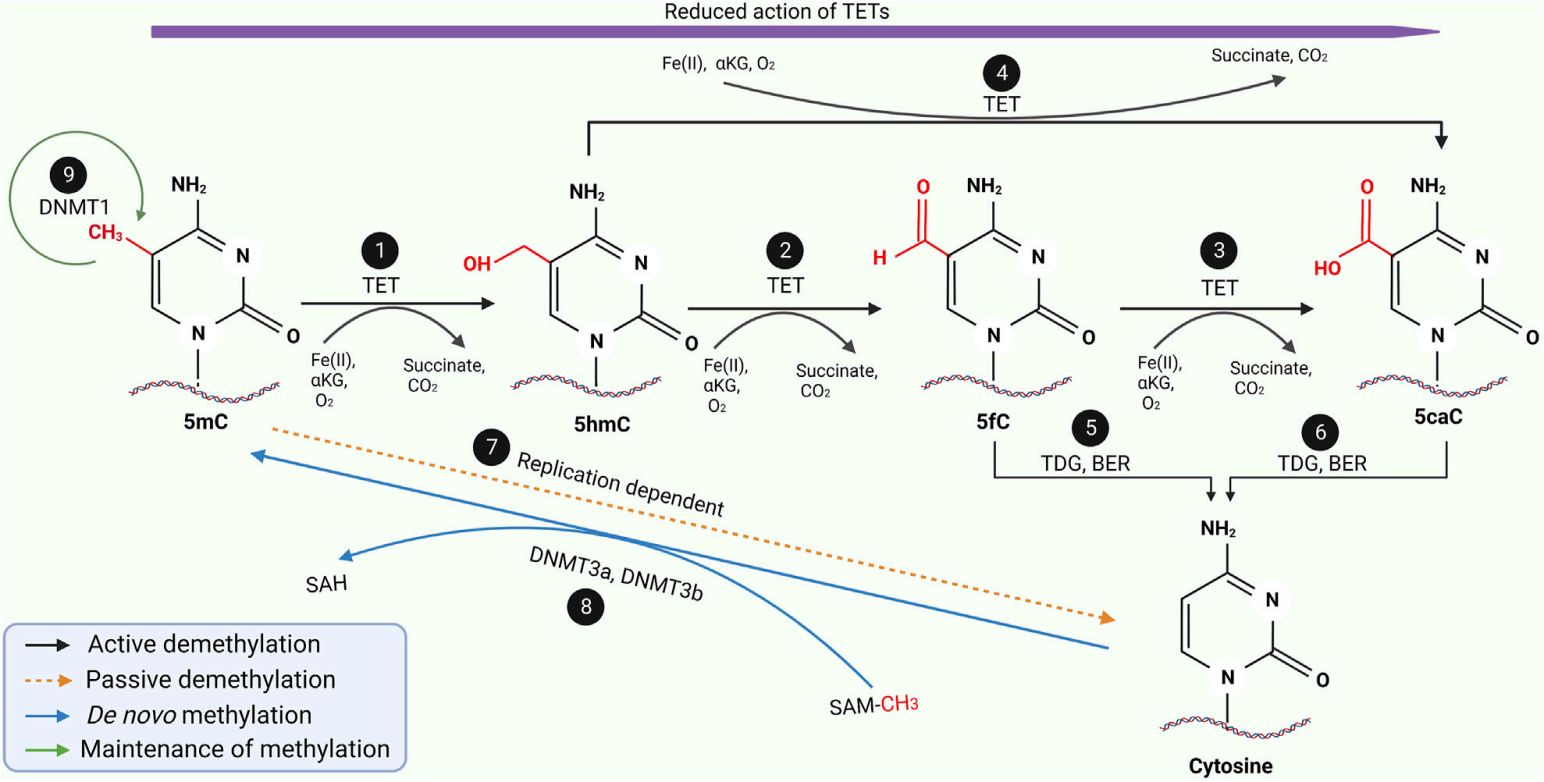
A schematic diagram of the entire methylation and demethylation processes of DNA. During replication, DNMT 1 ensures the maintenance of normal methylation patterns (9) while DNMT3a and DNMT3b mediates the *de novo* methylation of unmethylated cytosine during embryogenesis (8). During the active demethylation of 5mC by TET enzymes (1–6), iron (II) ions and α-ketoglutarate as well as oxygen molecule will be used as the substrate resulting in the byproduct of succinate and carbon dioxide. In the beginning the TET enzymes would catalyse the hydroxylation of 5mC to 5hmC (1) which is further oxidised into 5fC (2) followed by another oxidation into 5caC (3). Interestingly, 5hmC can also be directly oxidised into 5caC (4). During the entire active demethylation process, the oxidation potential of the TET enzymes would gradually reduce as represented by the purple arrow. 5fC and 5caC would then be excised by TDG leading to formation of an abasic site which would be repaired by BER to form regular unmethylated cytosine residue as shown in (5) and (6) respectively. Through a replication-dependent process, 5mC would be passively demethylated to unmethylated cytosine (7). Adapted and modified from (30, 31). Abbreviations: DNMT, DNA methyltransferase; TET, Ten-eleven translocases; TDG, Thymine DNA glycosylase; BER, Base excision repair; 5mC, 5-methycytosine; 5hmC, 5-hydroxymethylcytosine; 5fC, 5-formylcytosine; 5caC, 5-carboxycytosine.

DNA methylation is a reversible process whereby demethylation occurs, ensuring a gene does not remain repressed permanently. While not as well elucidated as methylation, it is known the methylated sequences can either be demethylated passively or actively, as illustrated in Figure 3 (32-34). Passive demethylation is known to be a replication-dependent process. In contrast, active demethylation is the removal of the methyl group by the dissolution of the carbon-carbon bond by methylcytosine dioxygenases known as Ten-eleven translocation (TET) family proteins (TET1, TET2 and TET3) as summarised in Table 2.

**TABLE 2:** Structure and function of Ten-Eleven Translocation proteins (TETs). Adapted from (19, 32-34).

Component		TET 1	TET 2	TET 3
Structure	N-terminal	Contains CXXC domain	Does not contain CXXC domain	Contains CXXC domain
	C-terminal	Contain DBHS domain, cysteine-rich domain and binding sites for Fe (II) and α-ketoglutarate cofactors forming the core catalytic domain		
Demethylation	Active	Catalyse oxidation of 5mC to oxidised methylcytosines which is excised by TDG to form abasic sites. Abasic sites will eventually form unmethylated cytosines through BER		
	Passive	Formation of 5hmC from TET enzymes activity has been proposed to contribute to passive demethylation		
Expression in cells		Highly expressed in embryonic stem cell blastocysts and primordial germ cells	Highly expressed in embryonic stem cells, blastocysts and during differentiation	Highly expressed in blastocysts as well as differentiated cells such as oocytes, zygotes, and neurons

CXXC, Cysteine-rich zinc ion binding domain; DBSH, *Drosophila* behaviour human splicing; TDG, thymine DNA, glycosylase; BER, base excision repair; 5mC, 5-methylcystosine; 5hmC, 5-hydroxymethylcytosine.

### Mitochondrial Epigenetics

Over the past half a century, there have been conflicting findings regarding mtDNA methylation (35). However, since the identification of mitochondrial-localised DNMT1 (mtDNMT1) and the presence of 5mC and 5hmC in mtDNA, more studies have supported the existence of mtDNA methylation (36). Some had indicated the existence of other epigenetic modifications, such as PTMs and ncRNAs in the mitochondria.

#### Mechanism of mtDNA Methylation

The primary methyl donor for mtDNA methylation is also S-adenosyl methionine which is imported into the mitochondria through a carrier (37). The DNMTs will catalyse the transfer of a methyl group from the S-adenosyl methionine onto the C5 of the cytosine nucleotides in the mitochondria. Among the many methyltransferases, only DNMT1 was reported to be involved in mtDNA methylation (36). While there are contradicting discoveries on the isoform responsible for mtDNA methylation, mtDNMT1 is the only DNMT catalytically active in mtDNA, whereby it initiates mtDNA methylation by binding to it (38). This interaction was observed in other investigations involving mouse embryonic fibroblast, human brain cells and human colon carcinomas (39, 40).

DNMT3a and DNMT3b were identified in the protein fraction of mitochondria of specific tissues (28, 41). DNMT3a was reported in excitable tissues such as neurons, skeletal muscle and embryonic stem cells, while DNMT3a and DNMT3b were found in breast cells (28, 41). Also, both global and regional mtDNA methylation was reduced in DNMT3a and DNMT3b knockdown studies, suggesting that the levels of these enzymes in the mitochondria may also contribute to mtDNA methylation of those specific tissues (28). While the functions and mechanisms of these enzymes are not fully elucidated, it is evident they can modulate the methylation pattern of mtDNA, and these patterns are specific for different cell types.

TET enzymes were found in mouse neuronal mitochondria indicating their possible role in regulating mtDNA methylation. TETs were demonstrated to be present in mtDNA with differing degrees of abundance of 5hmC depending on the cell type (42, 43). However, the overall levels of 5hmC are still lower than 5mC, with 5mC being the primary state of mtDNA methylation. It should be noted that while TETs were found to associate with 5hmC in mouse neuronal mitochondria, there is no definite evidence of their presence in other tissues (44). Therefore, it was suggested that there might be another mechanism for generating 5hmC in mtDNA.

In recent years, more groups have identified a high frequency of non-CpG methylation in mtDNA (45, 46). Bellizzi et al. (46) reported that most methylation patterns of human mtDNA were found to be present on cytosine residues outside of CpG dinucleotides with a higher degree of methylation on the L-strand and similar results were demonstrated in mouse mtDNA. Furthermore, high levels of N6-methyldeoxyadenosine (m6A) methylation were also discovered in mammalian mtDNA, whereby a report stated that the levels of m6A methylation of mtDNA were 1300-fold higher compared to nDNA. The higher levels of m6A methylation could have suggested that non-CpG methylation is more prominent in mtDNA than nDNA. Some theorised the possibility of adenine being the predominant site for mtDNA methylation (47, 48).

#### Impact of mtDNA Methylation

Since the first time mtDNA methylation was described, multiple pieces of evidence have indicated the impact of mtDNA methylation on several mitochondrial functions (48-52). Among the different regions, D-loop is the most well-annotated (51, 53-55).

Harbouring the origin of replication of heavy-strand and the promoters of both the heavy-strand and light-strand, methylation of the D-loop region will influence mitochondrial gene expression and mtDNA replication (53, 56). Mitochondrial gene expression has been reported to be impacted by D-loop mtDNA methylation *via* regulation of the binding and bending of mtDNA by mitochondrial transcription factor A. A higher degree of D-loop methylation would interrupt mitochondrial transcription factor A binding, leading to decreased transcription levels of mitochondrial genes, which would eventually result in significant changes in other mitochondrial functions (48, 57). Conversely, hypomethylation of the D-loop would increase gene expression (57). This was supported by several colorectal cancer cells investigations (51, 58, 59).

Replication of mtDNA which determine mtDNA copy number is another process hypothesised to be mediated by mtDNA methylation. This hypothesis was supported by other studies that reported alterations in the levels of mtDNA copy number in response to the upregulation and downregulation of D-loop methylation (51, 55). Investigations have indicated methylation levels, and mtDNA copy number is negatively correlated, whereby hypomethylation of mtDNA resulted in increased mtDNA copy number, and the inverse occurred for hypermethylation. Some groups reported that the hypermethylation at the promoters of the HSP on the D-loop of the diseased cells led to reduced mtDNA copy number compared to normal cells (40, 55). Meanwhile, an increased level of mtDNA copy number with hypomethylation of the D-loop region was reported in multiple colorectal cancer studies (51, 58, 59). Thus, this may explain the altered mitochondrial biogenesis of the diseased as it is dependent on the mtDNA copy number.

As for the coding regions, the impact of mtDNA methylation on them remains unknown as studies on the differential methylation in these regions remain largely unexplored. Recently, Mposhi et al. (60) showed an increased mtDNA methylation at the *CYTB* gene of the skeletal muscle of myopathy patients (60). The increased methylation was negatively correlated with the adenosine triphosphate production of the skeletal muscle of the myopathy patients. Another work showed hypermethylation at the mitochondrially-encoded *ND6 (MT-ND6)* gene resulted in impaired oxidative phosphorylation (52). However, more evidence is required to elucidate the exact correlation between methylation at the coding region and its expression.

In summary, methylation of the D-loop region impacts mitochondrial gene expression, whereby hypermethylation of the D-loop results in decreased gene expression levels and *vice versa*. Replication of mtDNA is also affected in which mtDNA copy number levels are inversely correlated to methylation leading to altered mitochondrial biogenesis. While the impact at the coding regions is not fully elucidated, studies indicate a negative correlation between methylation levels and its expression.

#### Post-Translational Modifications and Non-Coding RNAs

Other epigenetic modifications like PTMs and ncRNAs have been proposed to exist in the mitochondria. Similar to nDNA, PTMs in the mitochondria were found to modulate mitochondrial gene expression through alteration in mitochondrial transcription levels. PTMs in the mitochondria involve mitochondrial proteins such as nucleoid proteins which are integral in the organisation and functioning of the mitochondria (61). Among the various types of PTMs, acetylation and phosphorylation of nucleoid proteins have been indicated in multiple studies (61-64). Mitochondrial transcriptional factor A, a significant nucleoid protein in the maintenance of mtDNA, was found to be post-translationally modified *via* acetylation of the lysine residues and phosphorylation of the serine residues (62, 64). These two PTMs disrupted mtDNA packaging as the binding affinity of the nucleoid protein to mtDNA was reduced. Thus, this led to an alteration of the replication and transcription of mtDNA.

The ncRNAs involved in mitochondrial epigenetics are predominantly long ncRNAs (lncRNAs) and microRNAs (miRNAs). Both nuclear and mitochondrial genomes encode these ncRNAs, but it is uncertain whether mtDNA-encoded ncRNAs resulted from nuclear mitochondrial DNA or were transcribed in the mitochondria (65, 66). Regardless of their origins, ncRNAs were shown to modulate mitochondrial gene expression, and cellular and biological processes (67). Several lncRNAs identified were involved in both mitochondrial gene expression and cellular processes. They contained complementary sequences to mitochondrial ribosomal RNA and acted as retrograde signalling molecules critical to mito-nuclear crosstalk (68). Others, such as lncRNAs of *MT-ND5, MT-ND6* and *MT-CYB,* were found to form intermolecular duplexes with their respective mRNAs suggesting their roles in stabilising and regulating their mRNAs (65). Although the exact function of these lncRNAs is yet to be fully elucidated, it is apparent that they are involved in epigenetic regulation of mitochondrial gene expression.

As for miRNAs, they were mainly encoded by nDNA and translocated into the mitochondria, but while the mechanism is unknown, some miRNAs were encoded by mtDNA directly (69, 70). Most miRNAs were shown to downregulate mitochondrial protein expression *via* binding to the target mRNA leading to inhibition of translation or degradation of target mRNA. However, there was evidence that miRNAs could upregulate and downregulate protein expression at transcription and translation levels (70). For example, on a transcriptional level, *miR-2392* was shown to inhibit mtDNA transcription, while *miR-1* upregulates *MT-CO1* transcription (71, 72). On a translational level, *miR-181c* was found to downregulate *MT-CO1,* while *miR-21* upregulated *MT-CYB* protein expression (73, 74). While the mechanism and role of these miRNAs in the mitochondria have not been fully annotated, it is evident that they do play a part in mitochondrial gene expression and, subsequently the overall function of the mitochondria.

In short, other epigenetic modifications, such as PTMs and ncRNAs, could regulate mitochondrial gene expression either on a transcriptional or translational level. PTMs of nucleoid proteins could modulate the replication and transcription of mtDNA. Meanwhile, mitochondrial gene expression was altered by ncRNAs at both transcription and translation levels, which subsequently resulted in the regulation of other mitochondrial cellular and biological processes.

## Mitochondrial Epigenetic: Association With T2DM

Epigenetics, particularly nDNA methylation, is an established field with evidence showing the effect of nDNA methylation on T2DM as well as the impact of T2DM on nDNA methylation. As for the association between mtDNA methylation and T2DM, most of the understanding of their relationship is from indirect inference either through nDNA methylation-T2DM studies or nDNA methylation-obesity studies (75-77). However, in recent years, there have been studies directly addressing the correlation of mtDNA methylation with T2DM.

In one experiment, hypermethylation of the *MT-ND6* at the L-strand was observed in multiple tissues. The increased methylation was negatively correlated with its transcription, whereby T2DM patients showed a significant reduction in *MT-ND6* expression (52). This was further supported by the analysis of the peripheral leucocytes of T2DM patients in whom a significantly reduced *MT-ND6* level prevailed in response to its increased methylation compared to healthy subjects. Cao et al. (52) postulated that the decreased expression of *MT-ND6* may have indirectly contributed to the insulin resistance of T2DM, as *MT-ND6* is an essential aspect of the oxidative phosphorylation pathway (52). This was supported by the finding that both *MT-ND6* knockout mice and mice subjected to a diet that led to methylation at *MT-ND6* induced by DNMT1 had a higher likelihood of developing systemic insulin resistance than the control (52). Hence, it can be proposed that mtDNA methylation impacts the development of T2DM, as insulin resistance is a critical component in T2DM pathogenesis.

Zheng et al. (75) found that the degree of methylation at the D-loop affected the mtDNA copy number of the subjects. For the insulin resistance subjects, an increased D-loop methylation and reduction in the mtDNA copy number of their peripheral blood cells was observed compared to the insulin-sensitive subjects (75). The same was also observed in the obese group with significantly increased D-loop methylation and decreased mtDNA copy number compared to the lean group (75). The findings of this study corroborated with results from other diseases, which also showed a negative correlation between D-loop methylation and mtDNA copy number.

In the case of diabetic retinopathy, retinal cells obtained from cells cultured in high glucose and human donor showed a higher D-loop methylation at their retinal mtDNA compared to the control. Meanwhile, the cultured retinal cells also showed increased methylation at the *MT-CYB* region, but not as high as in the D-loop region (78). The hypermethylation of the mtDNA in response to the disease was supported by the increased expression of DNMT1 in their retinal microvasculature. It was also found that the transcripts of several mtDNA genes were reduced in response to the increased D-loop methylation in diabetic human donors compared to the control (78). This suggests that D-loop methylation does regulate mtDNA gene expression in T2DM. These findings indicate that mitochondrial epigenetics does have an impact on the development and progression of T2DM.

In summary, it appears that mtDNA methylation plays a role in the development and progression of T2DM. Hypermethylation of the coding region results in decreased expression due to reduced transcription levels in T2DM (52). Increased D-loop methylation would also lead to reduced mtDNA gene expression in T2DM (78). In addition, methylation of the D-loop region is negatively correlated with mtDNA copy number (75). In turn, this would alter mtDNA replication and, subsequently mitochondrial biogenesis (64).

## Challenges and Limitations

One of the main limitations of mitochondrial epigenetics study is the isolation of pure mitochondria from cells and clinical samples due to their small size and low abundance. According to Lampl et al. (79), the conventional approach to mitochondria isolation involves multiple centrifugation steps at different speeds (79). Thus, it is a very tedious procedure, limiting the number of samples that can be processed at one time. Regardless of the conventional method or commercial kit, a large amount of the initial sample is required for mitochondria isolation. This is not feasible in clinical settings if only a limited amount of blood or tissue is collected.

Secondly, mtDNA can be isolated either from pure mitochondria or cells. Isolation of mtDNA from pure mitochondria will be ideal for epigenetics study. However, due to the limited amount of mtDNA that can be obtained from pure mitochondria, it is usual to opt for the isolation of mtDNA from cells that contain a mixture of nDNA and mtDNA. This may result in bias involving amplification during the enrichment process, as the higher abundance of nDNA would mask mtDNA during amplification.

Furthermore, identifying mtDNA methylation itself is also challenging due to technical limitations. The gold standard of mtDNA methylation work is bisulphite sequencing, whereby bisulphite conversion coupled with PCR amplification are essential steps for the sequencing (35). However, amplification bias towards a highly methylated region may occur, resulting in inaccurate levels of methylated mtDNA. Incomplete bisulphite conversion or over-conversion of 5mC due to the small size and circular structure of mtDNA also affects the accuracy of the sequencing of mtDNA methylation (80). Hence, linearisation of mtDNA and good primer design are vital for bisulphite sequencing of mtDNA.

Currently, as there are contradicting views on the presence of methylated mtDNA, multiple sequencing methods are necessary to confirm the mtDNA methylation status (48, 80-82). A single targeted sequencing method may be insufficient to obtain a full methylation profile of the mtDNA. Targeted mtDNA methylation sequencing using Next-Generation Sequencing should be complemented with pyrosequencing to get a clearer view of mtDNA methylation profile.

Additionally, the technical limitations of mtDNA methylation studies are also observed in the non-CpG regions. Most of the commercially available bisulphite conversion kit and primers are biased toward CpG methylation, in which non-CpG nucleotides are usually assumed to be unmethylated (83). Hence, nanopore sequencing technology which does not rely on bisulphite conversion or PCR amplification, may be a better method for both CpG and non-CpG methylation studies (84, 85). However, more advancements in its software and chemistry are required before it can be routinely used for mtDNA methylation studies. The sequencing results could vary depending on the software used due to the different processing capacities and embedded datasets of each tool. While the chemistry of Nanopore eliminates DNA degradation from bisulphite conversion, it has difficulty detecting methylation of CpG sites which are in proximity and require higher DNA input than bisulphite sequencing (86). Also, it has a lower throughput and accuracy than short-read Next-Generation sequencing (87).

## Conclusion

Mitochondrial epigenetics is a challenging field mainly due to the small size and low abundance of the mitochondria, and its complex network with other cellular organelles. Nonetheless, studies supported the essential role of mitochondrial methylation in modulating mitochondria function. Hypermethylation at the D-loop region is associated with decreased mtDNA copy number of T2DM. Meanwhile, hypermethylation at both the D-loop and coding regions correlates to reduced gene expression. Overall, our finding shows that mtDNA methylation regulates T2DM pathogenesis. Understanding the mitochondrial epigenetics profile may aid in tracing T2DM pathogenesis and evaluating intervention efficacy in T2DM treatment.

## Abbreviations

DM, Diabetes mellitus; T2DM, Type 2 Diabetes mellitus; mtDNA, mitochondria DNA; D-loop, displacement loop; nDNA, nuclear DNA; CH_3_, methyl group; CpG, cytosine-phosphate-guanine (and so on for CpA, CpT and CpC); 5mC, 5-methylcytosine; DNMT, DNA methyltransferase; TET, Ten-eleven translocation methylcytosine dioxygenase; 5hmC, 5-hydroxymethylcytosine; mtDNMT1, mitochondrial-localised DNMT1, post-translational modification, PTM; non-coding RNA, ncRNA; messenger RNA, mRNA; long ncRNA, lncRNA; microRNA, miRNA; C5, fifth carbon, N6-methyldeoxyadenosine; m6A; PCR, polymerase chain reaction; *MT-CYB*, mitochondrially-encoded cytochrome b; *MT-ND*, mitochondrially-encoded nicotinamide adenine dinucleotide-hydrogen dehydrogenase; *MT-CO1*, mitochondrially-encoded cytochrome c oxidase 1.
